# 10 tips for clinical educators in designing and delivering learning experiences to improve clinical reasoning for medical students.

**DOI:** 10.12688/mep.21363.1

**Published:** 2025-11-18

**Authors:** Kelvin Le, Charlotte Deng, Khang Duy Ricky Le

**Affiliations:** 1 Melbourne Medical School, The University of Melbourne Faculty of Medicine Dentistry and Health Sciences, Melbourne, Victoria, Australia; 2 School of Medicine, Deakin University Faculty of Health, Geelong, Victoria, Australia; 3 Department of Medical Education, The University of Melbourne Faculty of Medicine Dentistry and Health Sciences, Melbourne, Victoria, Australia; 4 Department of General Surgical Specialities, The Royal Melbourne Hospital, Parkville, Victoria, Australia

**Keywords:** Clinical reasoning, medical students, medical education, health professionals’ education, medical curriculum.

## Abstract

**Background:**

Clinical reasoning processes involve gathering and interpreting information, creating differential diagnoses and testing hypotheses to inform and guide patient management. Effective clinical reasoning is an essential graduate outcome for medical students to ensure safe and efficient care of patients. In the clinical setting, a large proportion of hospital-related adverse events are attributed to errors in cognitive processes rather than knowledge, including diagnostic reasoning and decision-making. Teaching clinical reasoning is challenging due to its implicit nature, typically relying on internal thinking processes, pattern recognition and the use of prior clinical experiences. Current conventional teaching relies on student-driven application of clinical reasoning during their rotations as part of a hidden curriculum, which can be highly variable, unstructured, non-standardised, with limited oversight from faculty and with few opportunities for feedback. Furthermore, current barriers exist, including difficulties in teaching and assessing clinical reasoning. Due to this, many educators and faculty agree upon the significance of embedding explicit teaching and assessment of clinical reasoning into the curriculum, however the best approach remains poorly characterised.

**Methods:**

A narrative synthesis was undertaken from the current literature and the authors’ collective experience. The synthesis distils this information into ten practical tips to guide design, integration and innovation of clinical reasoning teaching in medical education.

**Results:**

Ten tips were identified to support educators’ efforts in embedding clinical reasoning into curriculum design and teaching. Together, these ten tips promote explicit, reflective and contextual clinical reasoning learning within the contemporary medical curriculum.

**Conclusions:**

Clinical reasoning requires deliberate, longitudinal and student-centred approaches that are integrated within authentic situated learning experiences. The ten tips provide avenues for more evidence-based adoption of effective learning environments that focus on clinical reasoning skills development.

## Introduction

Clinical reasoning (CR) is a cognitive process that involves gathering and interpreting information to generate hypotheses about diagnoses of the patient to guide management ^1, 2^. Although the medical curriculum is designed with the purpose of preparing graduate medical students to practice safely, CR is a crucial aspect of medical practice that is often inexplicitly addressed, difficult to teach and at times, neglected ^3– 6^. In 2015, the United States (US) Institute of Medicine reported that diagnostic errors contributed to approximately 10% of patient deaths and 17% of hospital adverse events ^7^. Importantly, it is posited that such errors are not typically the consequence of inadequate theoretical knowledge, but rather reflect flawed CR processes. CR however continues to be underemphasised and inconsistently integrated into formal medical curricula. This article presents 10 tips for clinical educators in designing and delivering learning experiences to improve CR.

### Tip 1: Embedding clinical reasoning explicitly into the curriculum

Assessment of history taking, examination skills and formulation of differential diagnoses from clinical findings are typically assessed in silos, which does not reflect the CR processes of the workplace. Having this non-standardised approach presents many challenges as CR processes are largely implicit, multi-step, experience-dependent, and difficult to process, which may result in cognitive overload ^8^. Additionally, student learning on placement is unstructured, variable and with limited available feedback and opportunities to critically review their CR ^3, 9, 10^.

Many propose CR teaching should occur in a longitudinal manner throughout the journey of medical education ^3, 11^. That is, rather than having the hidden expectation that students should develop their CR progressively, it is recommended to incorporate dedicated teaching of CR through structured activities and assessment ^12^. Important considerations include using evidence-based pedagogy to provide structural frameworks for curriculum design and implementation of explicit CR teaching ^12^. An example is provided by Choi *et al.*, who utilised experiential learning theory to explicitly incorporate CR teaching into a first-year medical curriculum. The authors demonstrated high rates of complete problem representation. Furthermore, a high number of students found improved 1) synthesis of medical knowledge, 2) diagnostic reasoning, 3) self-efficacy in CR processes and 4) awareness of cognitive biases ^13^.

### Tip 2: Constructive alignment

Constructive alignment (CA) refers to careful formulation of intended learning outcomes, followed by the design of aligned teaching/learning content and assessment ^14^. This includes design of learning outcomes to match clinical learning and assessment to facilitate the development of graduate outcomes ^6, 12, 15^. For learners, CA provides meaningful, goal-directed education by improving the clarity and understanding into what is expected and how learning activities and assessment are established to support development of work/graduate-specific skills ^6, 15, 16^. Studies have highlighted positive outcomes with course designs following principles of CA ^17, 18^. For example, Stickley recently demonstrated the application of CA in the development of four CR-related courses for physiotherapy students ^19^. This framework involves a 3-phase, 12-step process that encompasses identifying important learning outcomes, selection of active teaching and learning strategies, and incorporating feedback and assessment through a series of multimodal teaching strategies and examination processes. First year student feedback from this course design demonstrated improved confidence in formulating clinical decisions.

### Tip 3: High quality longitudinal clinical reasoning assessment

Multimodal forms of longitudinal assessment have shown benefit in assessing multiple aspects of complex CR processes. These can be categorised into non-workplace based assessments (written assessments or oral presentations), assessments in simulated clinical environments and workplace-based assessments ^19^. Assessing CR involves incorporating specific assessments that examine students’ diagnostic aptitude holistically to ensure the transfer of skills into practice. On the other hand, assessments that examine a specific part of the CR process can allow students to train these skills in broad contexts. In an ideal curriculum, incorporation of both ‘whole’ and ‘part’ task assessments form an effective foundation of teaching CR ^19^. When designing appropriate assessments for developing CR, Gordon *et al.* provided four main recommendations; 1) defining the construct of interest that is being assessed, 2) grounding assessments with specific cognitive theories to help students shape their understanding of CR and the rationale of assessments, 3) providing clarity of the mode of assessment delivery and its associated validation tools and scoring rubrics and 4) using specific frameworks to determine the validity and reliability of the assessment ^20^.

Furthermore, the scheduling and purpose of assessment is vital. Assessments can be both formative or summative, which draws on the concept of using assessments ‘for learning’ or ‘of learning’ respectively. A sole reliance on summative assessments can be suboptimal, creating a distinction between the educational process and assessing student knowledge/skill. Formative assessments serve as a learning tool for students, supplementing the educational process. More recently, the stance of designing assessments ‘as learning’ has been favoured by educators. This entails creating assessment structures that both judges examine the students’ ability and provide longitudinal and consistent opportunity for feedback and learning ^21– 23^.

### Tip 4: Engaging in specific and deliberate feedback processes

Feedback describes the process by which information delivered to compare a student's performance to that of expected graduate standards, with the goals of future sustainable improvement. As an active process, feedback bridges theoretical teaching with practical application, and is optimised through collaboration and co-creation of goals ^24– 26^. In medical education, feedback processes have been demonstrated to enhance the progressive development of knowledge and clinical performance, help assess current performance, improve self-awareness, support inherent motivation for learning and provide actionable plans for improvement ^27^.

According to Carless and Boud, student literacy is essential in accessing and making feedback useful. This literacy involves 1) appreciation of the value of feedback; 2) continually making judgements about their own work and the work of others; 3) managing affect and maintaining emotional equilibrium; and 4) taking action after receiving feedback ^25^. Therefore, feedback should explicitly align student goals and motivations with external objectives ^28^. This is relevant for teaching CR. Part of this is to ensure collaboration between student and assessor, and establish clarity in the feedback process ^26, 27, 29^. Several feedback models have been established including the Pendleton Rules, the one-minute preceptor, the SET-GO model, R2C2 and ALOBA model ^27^. Common aspects that enhance clarity for the students demonstrated in these models can be categorised based on student-driven, assessor-driven and mutual collaborative phases.

- Student-driven phases include creating dedicated time for students to reflect on the strengths and limitations of their own performance, alongside mutual exploration of the rationale behind decisions and choices made.
- Assessor-driven phases involve tailored and evidence-based input from the assessor that bridges theory with practical application aimed to improve and supplement student learning. With CR in mind, this includes a focus on outcomes, performance, transparency on how decisions were made, highlighting gaps in thinking, encourage alternative approaches and prompt justification of choices.
- Mutual collaborative phases involve discussion and co-creation of actionable goals that can improve future performance and enhance student motivation ^27, 28^.

Feedback should also be delivered in a timely manner and in a safe environment to optimise progressive, sustainable learning and student motivation.

### Tip 5: Leverage authentic case-based learning activities and simulation

Educators should integrate contextualised, case-based examples/simulations to better foster CR. In real-life clinical situations where presentations are often complex and multifaceted, purely didactic teaching fails to provide learners with directions on how to apply clinical knowledge ^30, 31^. Bleakley *et al.* posit that learning is contextualised and sensitive to its mediating factors, including the learners’ surrounding environment; this is congruent to Vygotsky's sociocultural theory of cognitive development which emphasises that learning is socially-mediated ^32, 33^.

CBL and simulation are active, learner-centred approaches that immerse students in realistic clinical problems to cultivate CR ^34^. In CBL, educators design authentic patient cases that unfold with progressive disclosure of the case, prompting students to apply CR processes throughout ^34, 35^. This format strengthens the relevance of pathophysiological concepts by situating them in the complex realities of practice and supports the transfer of theoretical knowledge to clinical contexts. Stimulation based teaching (SBT) is another authentic way to foster clinical skills by situating students as active participants to high fidelity and realistic clinical scenarios. SBT may involve a virtual reality environment (e.g. high-fidelity mannequin that mimics the patient) or other simulators that mirror real-life scenarios ^36, 37^. By situating learners in immediate clinical scenarios, SBT enhances skill learning, minimises errors, with co-benefits of increasing confidence and reducing anxiety ^38– 41^. A review on SBT by Elendu and Amaechi reported students were able to more quickly recognise and respond to critical conditions and made fewer clinical errors with SBT compared to more traditional training ^36^.

### Tip 6: Use active and relational learning strategies

Central to Vygotsky's sociocultural theory of cognitive development is the concept of zone of proximal development (ZPD), which contends that knowledge is co-constructed and distributed across and among people whereby learners achieve higher levels of understanding through collaboration ^32^. Skills central to CR therefore would be better fostered through active and relational learning, as would be expected of graduates who will commence work in multidisciplinary clinical environments. A systematic review on CR teaching identified active learning methods were superior to passive learning strategies like lectures, underscoring the need for pedagogical approaches that emphasise interaction and co-construction of knowledge ^4^. Strategies for facilitating active and relational learning include small-group discussions, think-pair-share, debates, and role-plays.

- Small group discussions including problem-based learning (PBL), CBL and team-based learning (TBL) create conducive environments for students to actively engage in open dialogue whereby critical thinking and clinical reasoning are openly fostered ^42^. In a randomised controlled trial amongst nursing students, problem solving was significantly improved for TL groups compared to control ^43^.
- Think-pair-share (TPS) is a cooperative and active learning strategy that involves individual deliberation on a given prompt, discussion with a partner, followed by whole group discussion ^44^. In an implementation of TPS amongst randomised groups of first-year medical students, TPS was identified with increased student participation and interaction, more equitable distribution of class participation, and improved quality of case discussion ^44^.
- Debates are a process of formulation of arguments and effective communication that aims to persuade the listeners to adopt a particular point of view. Therefore, it is a useful pedagogical tool for CR development where complex clinical scenarios lack a standardised answer and require multifaceted reasoning ^45^. A systematic review on the implementation of debates in medical education identified advantages including encouraging evidence-based learning, facilitating deliberate critical thinking and improving evaluation of clinical information ^45^.
- Role plays offer simulation for students to step into real-world clinical scenarios ^46, 47^. Huang *et al.* demonstrated that students who engaged in role play increased their CR skills in a mini-CEX scores, compared to control ^47^. Furthermore, Ronning and Bjorkly found increased reflection, insight, empathy and self-efficacy for students ^48^.

### Tip 7. Develop comprehensive digital spaces with diverse resources

The variability of learning styles among medical students is described and in recognition of this diversity, it is essential to curate a comprehensive range of CR activities that cater to these varied needs. Several inventories have been developed to categorise learning approaches. Examples include the Kolb Learning Style Inventory, which classifies learners as diverging, assimilating, converging, or accommodating; the VARK model, which identifies learners as visual, aural, read/write, or kinesthetic; and the Garsha-Reichmann Learning Type Scale, which distinguishes independent, avoidance, collaborative, dependent, competitive, and participant learning types ^49– 51^. With the proliferation of digital tools and digital spaces, new resources have emerged as popular adjuncts to traditional medical education. A survey of 1,083 medical students across two Australian medical schools revealed that in-person lectures, downloaded question banks, and medical apps were the most frequently used learning resources ^52^. Globally, similar trends have been reported, with increased use digital platforms such as “Meducation” and question banks like “PassMedicine”, accompanied by a decline in lecture attendance ^52^. These findings underscore the need for educators to remain aware of evolving student preferences and the rapid development of digital tools to align teaching with contemporary learning needs.

To address both the diversity in learning styles and variability in preferred learning modalities, educators should actively evaluate the unique needs of their student cohorts and provide a comprehensive suite of resources. For example, visual learners may benefit from videos, infographics, and diagrams; auditory learners from podcasts, narrated lectures, and discussion-based activities; read/write learners from textual resources, summaries, and note-taking; and kinesthetic learners from simulations, role-plays, and interactive clinical scenarios. Similarly, collaborative learners can thrive in team-based activities, while independent learners may prefer self-directed modules or digital question banks.

### Tip 8. Address cognitive biases

CR processes are formulated using available objective and subjective contextual data presented. As such, diagnostic and treatment errors can be largely attributed to inherent cognitive biases rather than deficits in medical knowledge ^53, 54^. Cognitive debiasing strategies that have been proposed to minimise errors in CR involve direct education to build insight into cognitive biases and developing tools that aid in improving accuracy to reduce the reliance on working memory ^55^.

***Developing insight and awareness of cognitive biases.*** Many studies have demonstrated the value of characterising the various types of cognitive biases and its impact on CR ^55^. Choi *et al.* demonstrated a well-received CR course, incorporating introductory didactic content of anchoring bias, availability bias, base-rate neglect, confirmation bias and representativeness bias, to prime student awareness of its existence and influence at the beginning of their CR course ^13^. In a similar vein, Chew *et al.*, designed 5 medical cases embedded with common cognitive biases and performed a quasi-experiment on final year medical students. Students educated about cognitive biases and debiasing performed significantly better compared to control ^56^. These studies highlight the role of specific education about common cognitive biases in improving CR. Furthermore, many cognitive biases are explained by the dual process theory (DPT). The DPT posits that diagnostic processes occur through two main types of thinking; type 1 (fast, automatic and intuitive pattern recognition) and type 2 (slow, analytical and deliberate) ^57^. Studies have highlighted the benefits of educating students about DPT to improve CR aptitude. Ainge *et al.* designed a clinical diagnosis assessment that explicitly develops dual process thinking for optometry students ^58^. Their study demonstrated a correlation between performance in this assessment to subsequent OSCE performance and examination ^58^.

***Supplementary tools and frameworks that aid in clinical reasoning.*** Providing students with supplementary tools and frameworks that aid in structuring CR processes are aimed at reducing errors made by cognitive biases as well as the reliance of the working memory. Many techniques have been mentioned in the literature, including the use of illness scripts, structured reflective approaches, mnemonics, schema-based reasoning, and case presentations.

- The script theory describes the organisation of knowledge and memory into discrete packages (‘scripts’), which is used to help apply previous experiences to current situations. For example, ‘illness scripts’ of conditions aid in stimulating processes of CR ^57, 59, 60^. Moghadami *et al.* demonstrated that fourth year medical students that were taught CR based on illness scripts had higher test scores based on symptoms and signs of the same diseases ^61^.
- Structured reflection involves a process of generating and refining differential diagnoses as more information is revealed. In doing so, this facilitates a constant reflective process aimed to develop CR ^62^. Mamede *et al.* demonstrated improvements in diagnostic accuracy scores in fourth year medical students that underwent the structured reflection approach compared to a single diagnosis or differential diagnosis approach ^63^.
- Mnemonics have also been proposed to aid in CR to help students create broader differential diagnoses ^3^. A study by Chai *et al.*, highlighted significant increases in differential diagnoses generation from students that used surgical sieves including VITAMIN CD compared to the control ^64^.
- Schema-based reasoning involves the process of grouping relevant diagnoses based on clinical features and pathophysiology. Blisset *et al.*, trialled a schema-based instructional approach, which revealed improved CR and retention of structured taught knowledge ^65^.
- Case presentation techniques are designed to provide structured CR processes. An example is SNAPPS, a 6-step process, which was demonstrated by Wolpaw *et al.* to lead to more concise case presentations, greater numbers of differential diagnoses and improved justifications and evidence of comparing and contrasting hypotheses ^66^.

### Tip 9. Co-design activities with students

Co-design engages students as partners to identify gaps, tailor activities, and create learning experiences that are more relevant and effective. The International Association for Health Professions Education (AMEE) ASPIRE initiative highlights student involvement as a hallmark of educational excellence, and AMEE’s Stakeholder Involvement in Co-Creation framework underscores that co-design benefits learners, teachers, and institutions by improving engagement and learning outcomes ^67, 68^.

Factors for success in curriculum co-design include proactive students, open-minded educators, and institutional support ^69^. However, challenges in curriculum co-designs still remain; students’ limited experience with course design, concerns about power dynamics, faculty fears of relinquishing control, and a lack of formal support and recognition from an institutional level ^67^. It is through conscious recognition of these challenges that educators can best facilitate optimal co-designing experiences that are effective and constructive.

### Tip 10. Model expert thinking

CR is an ingrained thought process relying on tacit knowledge that comes with experience, making it difficult for educators to articulate and for students to observe ^34^. To counter this challenge, educators should deliberately model their reasoning so that this cognitive process can be accessible to students ^70^.

The thinking-aloud technique involves the real-time verbalisation of thoughts while solving a clinical problem ^30, 70^. By exposing these otherwise automatic and subconscious processes, students can observe how expert clinicians link data, identify key features and avoid premature closure. In practice, this can be incorporated into case presentations by asking both students and clinicians to intermittently pause and articulate their CR.

Distillation of clinical heuristics into “thinking routines” is another approach. In an Australian study, Delany *et al.* identified complex clinical scenarios that students commonly found challenging, verbalised their own reasoning processes and condensed these into brief, repeatable prompts that students could apply in similar situations ^71^. Feedback from educators that employed thought routines in teaching reported students’ increased engagements in CR, including being better able to focus on received thinking routines include having structure that aids in on the spot thinking, and having a framework to work with to tease out important clinical details ^71^.

## Conclusion

CR is a fundamental graduate outcome for medical students to provide safe and efficient clinical care. However, CR processes are highly complex, implicit and often rely on prior experience. Without a solid foundation in practicing and developing CR, junior clinicians are more prone to creating preventable errors and adverse events. The tips presented in this article are aimed to help educators and faculty improve the design and delivery of CR teaching.

## Consent

Not applicable

## Data Availability

The data for this article consists of bibliographic references, which are included in the References section. Not applicable
