# Supplementary material for: 10 tips for clinical educators in designing and delivering learning experiences to improve clinical reasoning for medical students
Source: MedEdPublish (2016). 2026 Jan 18;15:272. Originally published 2025 Nov 18. [Version 4] doi: 10.12688/mep.21363.4 (PMC12796795; doi:10.12688/mep.21363.4)
Supplement: Peer review history

# Reviewer report: Reviewer response for version 3

**DOI:** 10.21956/mep.22992.r45104

**Published:** 2026-01-12

**Authors:** Yavuz Selim Kıyak

**Affiliations:** 1 Gazi University, Ankara, Turkey

Congrats for your hard work!

If evidence from practice is presented, are all the underlying source data available to ensure full reproducibility?

Not applicable

Is the topic of the practical tips discussed accurately in the context of the current literature

Partly

Are all factual statements correct and adequately supported by citations?

Partly

Are the conclusions drawn balanced and justified on the basis of the presented arguments?

Yes

Are arguments sufficiently supported by evidence from the published literature and/or the authors’ practice?

No

Reviewer Expertise:

Clinical reasoning, undergraduate medical education

I confirm that I have read this submission and believe that I have an appropriate level of expertise to confirm that it is of an acceptable scientific standard.

# Reviewer report: Reviewer response for version 2

**DOI:** 10.21956/mep.22959.r44808

**Published:** 2026-01-05

**Authors:** Christopher Runyon

**Affiliations:** 1 NBME, Philadelphia, USA

Thank you for the opportunity to review *10 tips for Clinical Educators in Designing and Delivering Learning Experience to Improve Clinical Reasoning for Medical Students*. The authors provide comprehensive guidance spanning curricular design, assessment methods, feedback processes, learning strategies, digital resources, and cognitive biases, while also appropriately acknowledging the content- and context-specific nature of clinical reasoning. I applaud the authors for their thoughtful selection of these 10 tips, as the landscape of clinical reasoning education offers numerous worthy candidates for inclusion.

 Given that this review will be published alongside the manuscript, I have aimed to provide constructive feedback that enhances the work's value to readers for the authors to consider. My comments are organized by tip and focus primarily on additional considerations, relevant literature, and opportunities to strengthen connections between the authors' recommendations. Rather than offering critique, I have sought to identify complementary perspectives and implementation considerations that may be useful to include.

**Tip 1 (Embedding CR explicitly in the curriculum) and Tip 2 (Constructure alignment):**

 Consistent with these goals would be the creation of programmatic objectives that explicitly address clinical reasoning instruction. As the authors note, there are often "hidden expectations" that students should develop CR through their coursework; establishing "Improving Clinical Reasoning" as an explicit programmatic goal can address this gap. Some work has been done in the United States to identify alignment between CR components and other standards (such as the AAMC Core Competencies), and similar alignment likely exists across other competency frameworks that the authors may wish to reference.

 The authors might also consider briefly addressing how institutions with already-established curricula can retrofit explicit CR instruction without major curricular overhaul, through mapping existing activities to CR competencies or identifying opportunities where CR can be made more explicit. Additionally, given the emphasis on constructive alignment, explicit CR objectives should naturally lead to explicit CR assessment criteria, creating transparency for students about what "good" clinical reasoning looks like while providing concrete feedback targets that address the "limited available feedback" challenge mentioned in Tip 1.

**Tip 3 (High Quality Longitudinal CR Assessment):**

 I appreciate that the authors identified that both 'whole' and 'part' CR task assessments are integral to supporting robust measurement of CR. To strengthen the longitudinal assessment approach, developmental trajectories that serve as guideposts for student achievement would be valuable. What does performance on these tasks look like for early, middle, and later students? How might assessment of the same task evolve as students develop their skills? These trajectories would complement the authors' emphasis on continued assessment and formative feedback.

 A similar assessment framework to consider (alongside Gordon et al.) is evidence-centered design. This validity framework begins by identifying claims about a student, then develops a theoretical model of what evidence would support those claims, and finally creates assessment tasks to generate that evidence. This approach mirrors the Gordon et al. process and may provide useful additional context for readers seeking frameworks for CR assessment design.

 Given the manuscript's emphasis on longitudinal assessment, the authors might also reference the programmatic assessment literature and the concept of a balanced assessment system, which emphasizes combining multiple assessment methods over time to build a comprehensive picture of CR competence. This aligns well with their multimodal approach and the "assessment as learning" philosophy. The authors could briefly acknowledge common implementation barriers to high-quality longitudinal CR assessment (e.g., faculty time, standardization across assessors, data management systems) and perhaps point to institutions or programs that have successfully implemented such systems as models for readers.

**Tip 4 (Engaging in Specific and Deliberate Feedback):**

 Feedback is a complex topic with substantial literature on best practices. Effective feedback is often content- and context-dependent, and not all guidance generalizes well across assessment contexts. Ultimately, the best feedback is feedback that students actually use. Related to this point—feedback should be timely, but equally important is providing timely opportunities to apply that feedback. Even excellent feedback becomes ineffective if students cannot use it or practice the skill soon after receiving it.

 The authors might also address what makes feedback for clinical reasoning distinct from feedback on more discrete skills. CR feedback often requires unpacking the student's thinking process and addressing both the outcome (diagnosis/management plan) and the reasoning pathway. This may require different approaches than feedback on technical skills or knowledge recall. Given the variety of feedback models presented and the complexity of providing effective CR feedback, the authors could acknowledge that educators may need specific training or faculty development to deliver high-quality CR feedback. This is particularly relevant since many clinical educators may not have formal training in feedback delivery for complex cognitive processes.

**Tip 5 (Leverage authentic case-based learning activities and simulation):**

 This tip addresses an important standardization-authenticity trade-off in clinical education. Workplace-based assessments provide students with authentic settings to practice their skills, but the patient encounters are variable and cannot be repeated. Non-workplace based assessments offer standardization but may lack authenticity. The authors identify that CBL and simulation-based teaching may provide a valuable middle ground, balancing authenticity while maintaining task standardization to ensure all learners receive comparable learning experiences.

 The authors mention progressive disclosure in CBL but could elaborate on how this feature specifically supports CR development by mimicking the iterative nature of real clinical encounters. Additionally, connecting this tip to Tip 4, simulation and CBL provide immediate, structured opportunities for the deliberate feedback processes previously advocated; students can apply feedback and practice CR skills repeatedly with similar cases. While the benefits of high-fidelity simulation are clear, the authors could briefly acknowledge resource considerations (cost, space, faculty time) and note that lower-fidelity approaches (written cases, standardized patients, virtual cases) can also effectively develop CR when thoughtfully designed. This would make the tip more accessible to educators working with varied resources.

**Tip 6 (Use active and relational learning strategies):**

 The authors' characterization of Vygotsky's zone of proximal development (ZPD) could be clarified. The ZPD traditionally refers to the gap between what learners can accomplish independently and what they can achieve with guidance or assistance, bridging skills they have already mastered and those they cannot yet perform alone. While the collaborative aspect of learning that the authors emphasize is consistent with ZPD, the key element is that someone in the collaboration (whether peer or instructor) possesses the skill being learned and can provide appropriate scaffolding.

 Given this scaffolding concept, the authors might address the facilitator's role in these active learning strategies. Effective small-group discussions, debates, and role-plays for CR development require skilled facilitation to ensure productive discourse, identify gaps in reasoning, and provide appropriate guidance (connecting back to the faculty development considerations in Tip 4). Additionally, while the authors provide excellent examples of active learning strategies, brief guidance on when each strategy is most appropriate for developing different aspects of CR would be valuable. Debates might be particularly suited for exploring diagnostic uncertainty and weighing evidence; role-plays may better address patient interaction and information gathering aspects of clinical reasoning.

**Tip 7 (Develop Comprehensive Digital Space with Diverse Resources):**

 This tip raises some concerns regarding the guidance provided. The learning style theories identified by the authors (Kolb LSI, VARK, and Garsha-Reichmann LTS) have utility for describing student learning preferences, but these theories largely lack strong empirical evidence to guide instructional design decisions. Studies examining learning style preferences have shown weak correlational relationships at best and, at worst, poor psychometric properties with limited practical utility for curriculum development.

 The authors' observation that students increasingly rely on online or third-party resources, often resulting in decreased class attendance, is accurate but warrants deeper consideration. While many online repositories exist to support student learning, it remains unclear whether these materials provide students with the foundational knowledge and structured CR development necessary for clinical competence.

 The authors appropriately identify that AI models may provide utility in developing materials for CR instruction or assessment. However, AI should be positioned as a tool to enable the other tips provided for CR instruction rather than as a standalone solution. Best practices for using AI in health professions education are still emerging, and the authors should emphasize the importance of verification processes to ensure AI-generated content aligns with current best practices in medicine.

**Tip 8 (Address Cognitive Biases):**

 While the authors appropriately address cognitive biases and dual process theory (DPT) as important factors in clinical reasoning errors, expanding this discussion to include the transtheoretical model of clinical reasoning would strengthen this section. The transtheoretical model, proposed by Parsons et al. (2024), integrates DPT with situativity theories (embodied cognition, ecological psychology, situated cognition, and distributed cognition) to provide a more comprehensive understanding of how clinical reasoning occurs both "in the head" and "out in the world." This framework acknowledges that while cognitive biases play an important role in diagnostic errors, reasoning is also significantly influenced by contextual factors, environmental affordances, and system-level interactions that extend beyond individual cognitive processes. Incorporating this model would naturally complement the authors' earlier emphasis on content- and context-specificity in clinical reasoning, reinforcing that effective CR instruction must address both individual cognitive factors and the complex environments in which clinical decisions are made.

**Tip 9 (Co-design Activities with Students):**

 The authors appropriately identify the benefits of co-design in improving educational relevance and effectiveness. An additional important advantage of co-design is sustained learner engagement with the materials. When students participate in creating learning experiences, they develop deeper investment in and ownership of their learning, which can enhance motivation and long-term engagement with clinical reasoning development throughout their training.

 The co-design process itself offers valuable educational benefits. Students must reflect on their own learning processes and identify what helps them reason effectively, fostering metacognitive development and self-regulated learning skills essential for clinical practice. Senior learners, who are closer to entering practice, can provide particularly valuable perspectives on which clinical reasoning challenges feel most authentic and relevant to their anticipated clinical roles, helping bridge the gap between educational exercises and real-world clinical demands (complementing the authors' earlier emphasis on authentic case-based learning in Tip 5). Finally, co-design creates opportunities for iterative improvement of clinical reasoning instruction based on direct student feedback, allowing educators to adapt more quickly to emerging challenges and respond to diverse learning needs across student cohorts.

**Tip 10 (Model Expert Thinking):**

 While the authors appropriately emphasize the value of modeling expert thinking, they might acknowledge an important challenge: experts often have such well-developed illness scripts that much of what was once deliberate, analytical (Type 2) thinking has become fast, automatic (Type 1) pattern recognition. This expertise paradox means that articulating one's reasoning process is itself a separate pedagogical skill that may require explicit faculty development. Educators need training not only in clinical reasoning but also in how to effectively externalize and deconstruct their automated cognitive processes for learners.

 Additionally, expert thinking should include not just correct reasoning pathways but also how experts manage diagnostic uncertainty, recognize when they might be wrong and how they recover from errors. Demonstrating these metacognitive processes helps students understand that clinical reasoning is iterative and that even experts must continuously monitor and adjust their thinking. Educators might also model how their reasoning process adapts to different clinical contexts, showing students how the same diagnosis might be approached differently depending on practice setting, available resources, or patient factors. This reinforces that expert thinking is flexible and context-dependent, connecting back to the situated nature of clinical reasoning discussed in earlier tips. Finally, the authors could suggest that students also practice thinking aloud during clinical encounters, not just observing faculty doing so. This reciprocal approach allows educators to identify gaps in student reasoning in real-time and provide immediate corrective feedback, creating a bidirectional learning opportunity that enhances both student development and faculty awareness of learner needs.

If evidence from practice is presented, are all the underlying source data available to ensure full reproducibility?

Not applicable

Is the topic of the practical tips discussed accurately in the context of the current literature

Yes

Are all factual statements correct and adequately supported by citations?

Yes

Are the conclusions drawn balanced and justified on the basis of the presented arguments?

Yes

Are arguments sufficiently supported by evidence from the published literature and/or the authors’ practice?

Partly

Reviewer Expertise:

Assessment, Clinical Reasoning, Artificial Intelligence, Cognitive Science

I confirm that I have read this submission and believe that I have an appropriate level of expertise to confirm that it is of an acceptable scientific standard.

## References

[1] : Beyond thinking fast and slow: Implications of a transtheoretical model of clinical reasoning and error on teaching, assessment, and research. *Medical Teacher* .2025;47(4) : 10.1080/0142159X.2024.2359963 665-676 10.1080/0142159X.2024.2359963 38835283

# Reviewer report: Reviewer response for version 2

**DOI:** 10.21956/mep.22959.r44777

**Published:** 2025-12-30

**Authors:** Yavuz Selim Kıyak

**Affiliations:** 1 Gazi University, Ankara, Turkey

The authors successfully addressed most of my concerns. In Tip 7, the first three sentences are about learning styles. As I stated in my previous report,these are considered as a myth in the scientific sense. People do have preferences, but there is no good evidence that teaching to a student’s preferred style makes them learn better. Therefore, the first three sentences in Tip 7 should be removed. They are also now irrelevant in the context of "Develop comprehensive digital spaces with diverse resources".

If evidence from practice is presented, are all the underlying source data available to ensure full reproducibility?

Not applicable

Is the topic of the practical tips discussed accurately in the context of the current literature

Partly

Are all factual statements correct and adequately supported by citations?

Partly

Are the conclusions drawn balanced and justified on the basis of the presented arguments?

Yes

Are arguments sufficiently supported by evidence from the published literature and/or the authors’ practice?

No

Reviewer Expertise:

Clinical reasoning, undergraduate medical education

I confirm that I have read this submission and believe that I have an appropriate level of expertise to confirm that it is of an acceptable scientific standard, however I have significant reservations, as outlined above.

# Response

**Published:** 2025-12-30

**Authors:** Khang Duy Ricky Le

**Affiliations:** School of Medicine, Deakin University Faculty of Health, Geelong, Victoria, Australia

We thank the reviewer for their comment. We have amended the tip 7 as suggested.

# Reviewer report: Reviewer response for version 1

**DOI:** 10.21956/mep.22880.r44700

**Published:** 2025-12-02

**Authors:** Yavuz Selim Kıyak

**Affiliations:** 1 Gazi University, Ankara, Turkey

This practical tips article provides a broad and practical overview of strategies to improve clinical reasoning (CR) in undergraduate medical education. It links applied teaching tips with established educational theories, such as constructive alignment, experiential learning, Vygotsky’s Zone of Proximal Development, dual process theory, and script theory. In addition to focusing on individual teaching sessions, it also mentions strengths related to curriculum design, assessment, feedback culture, and student partnership. This is a clear sign that the authors consider teaching as a whole, which should be appreciated.

 The article is well-written but I have two major and some minor recommendations.

**Major:**

1. In Tip 7, the authors follows the learning styles (visual / auditory / kinesthetic, VARK, etc.) myth. These are considered a myth in the scientific sense. People do have preferences, but there is no good evidence that teaching to a student’s preferred style makes them learn better. (Nancekivell, S. E., Shah, P., & Gelman, S. A. (2020). Maybe they’re born with it, or maybe it’s experience: Toward a deeper understanding of the learning style myth. Journal of Educational Psychology, 112(2), 221. Hattie, J., & O’Leary, T. (2025). Learning styles, preferences, or strategies? an explanation for the resurgence of styles across many meta-analyses. Educational Psychology Review, 37(2), 1-26.) After completely removing learning styles, I would choose to mention the possible use of AI and LLMs for teaching CR, as the tip includes digital aspects. There are some empirical studies that show effectiveness of using AI to teach CR, which you can use to support your statements.
2. The stated focus is medical students but many examples and studies are taken from other health professions such as nursing and physiotherapy. It is a richness rather than a problem but the issue is the inconsistency between the stated focus and the content. This needs to be explained (for example by stating that this is primarily for undergraduate medical curricula with cross-professional examples) or reframed.

**Minor**

1. It would be better if the first part of the article, just before the tips, includes the fact that CR is not a generic skill, as this is so influential on how you should teach, it is domain-, content-, and context-specific, by citing an appropriate source, such as: Holmboe, E. S., & Durning, S. J. (2014). Assessing clinical reasoning: moving from in vitro to in vivo. Diagnosis, 1(1), 111-117.
2. Based on the theory someone adopt, the approach to teach CR would change. Therefore, if possible, it would be better if the authors provide their theoretical stance to approach to giving tips, or briefly mentioning the theory in each tip as they already have done in some of the tips.
3. Some specific tips are the tips for general good teaching rather than CR. In those instances, add a clear relationship on how it specifically supports CR.
4. The cognitive bias section focuses on positive findings and does not mention challenges or mixed results in debiasing research. Debiasing and CR teaching are necessary but not sufficient to eliminate diagnostic error. System and organizational factors also play an important role. This fact should be recognized.
5. Some tips list many approaches (CBL, TBL, etc. ) with single supporting studies. Whenever it is possible, also refer to systematic/scoping reviews to provide a more comprehensive evidence.
6. “Stimulation based teaching” should be “Simulation-based teaching”.
7. Tip headings should be standard (“Tip 7.” or “Tip 1:”).

If evidence from practice is presented, are all the underlying source data available to ensure full reproducibility?

Not applicable

Is the topic of the practical tips discussed accurately in the context of the current literature

Partly

Are all factual statements correct and adequately supported by citations?

Partly

Are the conclusions drawn balanced and justified on the basis of the presented arguments?

Yes

Are arguments sufficiently supported by evidence from the published literature and/or the authors’ practice?

No

Reviewer Expertise:

Clinical reasoning, undergraduate medical education

I confirm that I have read this submission and believe that I have an appropriate level of expertise to confirm that it is of an acceptable scientific standard, however I have significant reservations, as outlined above.

# Response

**Published:** 2025-12-03

**Authors:** Khang Duy Ricky Le

**Affiliations:** School of Medicine, Deakin University Faculty of Health, Geelong, Victoria, Australia

We thank the reviewer for their comments, attention to detail and high quality feedback aimed at improving the quality of our manuscript. We have revised the mansucript according to this and are pleased and humbled to submit a revised version for review. Below are a summary of the changes made. For orientation, the reviewer comments are provided verbatim. 

**Major:**

1. **In Tip 7, the authors follows the learning styles (visual / auditory / kinesthetic, VARK, etc.) myth. These are considered a myth in the scientific sense. People do have preferences, but there is no good evidence that teaching to a student’s preferred style makes them learn better. (Nancekivell, S. E., Shah, P., & Gelman, S. A. (2020). Maybe they’re born with it, or maybe it’s experience: Toward a deeper understanding of the learning style myth. Journal of Educational Psychology, 112(2), 221. Hattie, J., & O’Leary, T. (2025). Learning styles, preferences, or strategies? an explanation for the resurgence of styles across many meta-analyses. Educational Psychology Review, 37(2), 1-26.) After completely removing learning styles, I would choose to mention the possible use of AI and LLMs for teaching CR, as the tip includes digital aspects. There are some empirical studies that show effectiveness of using AI to teach CR, which you can use to support your statements.**

We thank the reviewer for their comment. We have attended to this suggestion which is addressed in lines 190-196, 198. We have also added corresponding references 56 and 57 and deleted lines 200-207 to remove learning styles.

1. **The stated focus is medical students but many examples and studies are taken from other health professions such as nursing and physiotherapy. It is a richness rather than a problem but the issue is the inconsistency between the stated focus and the content. This needs to be explained (for example by stating that this is primarily for undergraduate medical curricula with cross-professional examples) or reframed.**

We thank the reviewer for their comment. We have attended to this suggestion which is addressed in lines 16-19

**Minor**

1. **It would be better if the first part of the article, just before the tips, includes the fact that CR is not a generic skill, as this is so influential on how you should teach, it is domain-, content-, and context-specific, by citing an appropriate source, such as: Holmboe, E. S., & Durning, S. J. (2014). Assessing clinical reasoning: moving from in vitro to in vivo. Diagnosis, 1(1), 111-117.**

We thank the reviewer for their comment and have addressed this in lines 5, 10-14, with the addition of reference 8 

1. **Based on the theory someone adopt, the approach to teach CR would change. Therefore, if possible, it would be better if the authors provide their theoretical stance to approach to giving tips, or briefly mentioning the theory in each tip as they already have done in some of the tips.**

We thank the reviewer for their comment. As mentioned, we have provided relevant learning theories, pedagogy and models for tips 2,3, 4, 5, 6, 7, 8. Tips 1, 9 and 10 are practical and therefore adding learning theory would not improve the discourse on integration, curriculum design or development. Therefore no changes have been made as doing so would be stretching the discussion with no clear benefit and render the manuscript more lengthy.

  

1. **Some specific tips are the tips for general good teaching rather than CR. In those instances, add a clear relationship on how it specifically supports CR.**

We thank the reviewer for their suggestion and have addressed CR more specifically in tip 2 (lines 46-47), tip 4 (89-91), tip 7 (lines 190-196 with references 56 and 57), tip 9: (lines 279-281)

  

1. **The cognitive bias section focuses on positive findings and does not mention challenges or mixed results in debiasing research. Debiasing and CR teaching are necessary but not sufficient to eliminate diagnostic error. System and organizational factors also play an important role. This fact should be recognized.**

We thank the reviewer for their comment and have addressed this in lines 268-275. Added references 74 and 75.

1. **Some tips list many approaches (CBL, TBL, etc. ) with single supporting studies. Whenever it is possible, also refer to systematic/scoping reviews to provide a more comprehensive evidence.**

We thank the reviewer for their comment. We have attended to the suggestion within the limitations of the evidence and the scope of our practical tips manuscript. Please see below the relevant comments as they relate to each tip.

1.
  - Tip 1: Referenced a systematic review in lines 33-34.
  - Tip 2 – couldn’t find another one that matches
  - Tip 3 : Referenced a scoping review in lines 58-59.
  - Tip 4 – not relevant
  - Tip 5 – already has a review paper so did not add another
  - Tip 6: Added a study for TBL in lines 151-153 (reference 45) and discussed these in general. Deleted specific study details in lines 153-155. Added another study for TPS (reference 47) lines 161-163.
  - Tip 7: AI and LLM scoping review and systematic review added lines 190-196, 198. Added references 56 and 57
  - Tip 8: Lines 243-244 added systematic review (reference 67) for illness scripts. Lines 247-249 and 252-254 added systematic review that included structured reflection. No relevant sources for mnemonics and schemas. Lines 264-265 (added reference 72).
  - Tip 9: not applicable
  - Tip 10: not applicable
2. **“Stimulation based teaching” should be “Simulation-based teaching”.**

We thank the reviewer for their comment. We have corrected to simulation-based teaching (line 128)

  

1. **Tip headings should be standard (“Tip 7.” or “Tip 1:”).**

We thank the reviewer for their comment have have amended the tip headings i.e. changed to Tip X: for consistency (lines 176, 208, 277, 292) **Below are additional changes that we have also  made to improve the quality of the manuscript.** References that were added

-
  - 8: Holmboe, ES, Durning SJ: Assessing clinical reasoning: moving from in vitro to in vivo. Diagnosis. 2014;1(1):111–117. 10.1515/dx-2013-0029
  - 45: Ozgonul L, Alimoglu MK: Comparison of lecture and team-based learning in medical ethics education. Nursing ethics. 2019;26(3):903–913. 10.1177/0969733017731916
  - 47: Kaddoura M: Think pair share: A teaching learning strategy to enhance students' critical thinking. Educational research quarterly. 2013;36(4):3–24.
  - 56: García-Torres D, Vicente Ripoll MA, Fernández Peris C, Mira Solves JJ: Enhancing clinical reasoning with virtual patients: a hybrid systematic review combining human reviewers and ChatGPT. Healthcare. 2024;12(22):2241. 10.3390/healthcare12222241
  - 57: Liu YM, Chou CC, Jaing TH, Okoli CT: Generative AI for clinical reasoning: A scoping review. Teaching and Learning in Nursing. 2025. 10.1016/j.teln.2025.08.008
  - 67: Si J: Strategies for developing pre-clinical medical students’ clinical reasoning based on illness script formation: a systematic review. Korean journal of medical education. 2022;34(1): 49–61. 10.3946/kjme.2022.219
  - 72: Sawanyawisuth K, Schwartz A, Wolpaw T, Bordage G: Expressing clinical reasoning and uncertainties during a Thai internal medicine ambulatory care rotation: does the SNAPPS technique generalize?. Medical teacher. 2015;37(4): 379–384. 10.3109/0142159X.2014.947942
  - 74: Tung A, Melchiorre M (2023). Debiasing and Educational Interventions in Medical Diagnosis: A Systematic Review. University of Toronto Medical Journal. 2023;100. 10.33137/utmj.v100i1.38937.
  - 75: Croskerry P, Singhal G, Mamede S: Cognitive debiasing 1: origins of bias and theory of debiasing. BMJ Quality & Safety. 2013;22(Suppl 2):ii58-ii64. 10.1136/bmjqs-2013-002387

In-text references that were changed due to inclusion of new references

-
  - Lines 25, 27, 29, 31, 34, 38, 41, 43, 46, 49, 50, 61, 66, 71, 80, 85, 89, 95-96, 97, 98, 100, 112, 119, 122, 124, 126, 131, 133, 136, 141, 151, 158, 161, 167, 170, 171, 172, 173, 174, 184, 188, 190, 211, 214, 216, 220, 223, 227, 229, 231, 241, 243, 247, 251, 258, 262, 267, 285, 287, 290, 294, 296, 298, 305, 309.

Other comments

-
  - Line 82 – syntax error, added ‘is’
  - Line 188 – syntax error, added ‘of’
  - Tracked changes of all the references numbers (no page number given)
